# The use of structured light plethysmography in assessing the outcome of lung reduction

**DOI:** 10.1186/1749-8090-10-S1-A359

**Published:** 2015-12-16

**Authors:** Ghazi Elshafie, Babu Naidu

**Affiliations:** 1 Heart of England NHS Trust, B95SS, Birmingham, UK

## Background/Introduction

Lung volume reduction surgery via endobronchial valves (EBV) insertion improves clinical outcomes and quality of life in selected patients with emphysema. The response to this intervention has been inconsistent because collateral ventilation prevents lobar atelectasis. Outcomes of this procedure are measured radiologically. But these radiological methods lack sensitivity and cannot identify non-responders immediately after surgery

## Aims/Objectives

Thus we evaluated the viability of a novel portable device to measure success/failure of EBV insertion by measuring the lobar atelectasis effect on dynamic chest wall motion.

## Method

Structured Light Plethysmography (SLP) measures chest wall motion using a light grid which is simultaneously 'seen' by a digital vision system. Measurements were made during quiet breathing, before and immediately after EBV insertion and for up to 3 days post operatively.

## Results

Three male COPD patients who underwent EBV LVR were assessed. One patient had a successful procedure as deemed by CT findings and clinical improvement. In this patient SLP detected a significant reduction in the chest wall motion on the valves receiving side compared to global chest wall motion immediately postoperatively that was sustained during subsequent measurements from 55 +/- 0.99 % pre-op to 48 +/- 0.60 % at 2nd post-operative day (P < 0.001). This was mirrored by an improvement in his Borg breathless score from 7 to 0. In the second patient the valves did not result in lobar reduction and there was no significant reduction in measured motion. In the third patient the valves had an immediate beneficial effect on chest wall motion but this was reversed by the development of a para-valvular leak.

## Discussion/Conclusion

SLP can detect immediate successes or failure of EBV LVR and may be a useful tool in monitoring and understanding benefits for this surgery.

